# Preliminary exploration of potential biomarkers for heart failure and bipolar disorder: an exploratory study based on bioinformatics

**DOI:** 10.3389/fpsyt.2025.1627105

**Published:** 2025-09-10

**Authors:** Wei Zhang, Na Li

**Affiliations:** ^1^ Department of Emergency, Hebei Provincial Hospital of Traditional Chinese Medicine, Shijiazhuang, China; ^2^ Department of Psychiatric and Psychological, Hebei Provincial Hospital of Traditional Chinese Medicine, Shijiazhuang, China

**Keywords:** bipolar disorder, heart failure, biomarkers, WGCNA, GSEA

## Abstract

**Background:**

Individuals with bipolar disorder (BD) exhibit a significantly increased risk of cardiovascular disease, yet the specific mechanisms linking heart failure (HF) and BD remain poorly understood. This study aimed to identify common potential diagnostic biomarkers associated with both conditions.

**Methods:**

Differentially expressed genes (DEGs) were analyzed separately in HF (GSE57338) and BD (GSE5389) datasets. Key module genes for each condition were identified through co-expression network analysis and intersected with DEGs to pinpoint candidate genes. Subsequently, a protein-protein interaction (PPI) network, receiver operating characteristic (ROC) analysis, and expression validation were employed to identify potential diagnostic biomarkers. Gene set enrichment analysis (GSEA) and drug predictions were also conducted. Clinical validation of biomarker expression was performed via quantitative polymerase chain reaction (qPCR).

**Results:**

A total of 44 candidate genes were identified as being associated with both HF and BD. Six potential diagnostic biomarkers (*UBE2E3, FZD2, EXT1, DCHS1, BMP4*, and *ALDH1A2*) were selected. These biomarkers were predominantly linked to the “cytokine-cytokine receptor interaction” and “ECM receptor interaction” pathways. Additionally, four potential drugs—VANTICTUMAB, RETINOL, HYDROCHLOROTHIAZIDE, and ATENOLOL—were identified as targets for these biomarkers. Expression trends of *FZD2, DCHS1, BMP4*, and *ALDH1A2* validated by qPCR were consistent with dataset findings.

**Conclusion:**

This study preliminarily explored the common molecular mechanisms between HF and BD, and identified 6 potential biomarkers for early detection, providing a solid theoretical basis for future research on HF and BD.

## 1 Introduction

Bipolar disorder (BD) is a severe mental disorder that typically first appears in adolescence or young adulthood (1). Studies have shown that the incidence rate of BD ranges from 1% to 3% (2, 3). The disease is mainly characterized by significant mood swings, neuropsychological deficits, and major changes in the physiological and immune systems. These changes may lead to dysfunction and are accompanied by a higher mortality rate (4, 5). BD includes three main phases: depressive phase, manic phase, and hypomanic phase. Patients experience recurrent episodes between these phases, going through periodic mood swings (6). Concurrently, societal advances, shifts in lifestyle, and an aging population have contributed to a significant rise in heart failure (HF) cases, placing considerable strain on public health (7, 8). Notably, research has demonstrated that individuals with severe mental illnesses, such as BD, schizophrenia, and major depression, carry a disproportionate cardiovascular disease (CVD) burden compared to the general population, resulting in a life expectancy reduction of approximately 20 years for these individuals (9, 10). These findings underscore the urgent need for targeted interventions to prevent cardiovascular mortality in these high-risk groups.

The heightened cardiovascular risk in BD can be attributed to several factors, including lifestyle choices, adverse effects of psychotropic medications, and shared genetic predispositions between severe psychiatric disorders and CVD (11, 12). Furthermore, patients with BD often exhibit autonomic nervous system dysfunction, leading to reduced heart rate variability compared to healthy individuals, which further elevates the risk of cardiovascular events (12). Psychological stressors, such as emotional fluctuations and heightened anxiety, are common among patients with BD and may exacerbate cardiac dysfunction through neuroendocrine pathways, intensifying HF symptoms (13). Recent studies have highlighted the significance of the heart-brain axis in regulating cardiac function, particularly in patients with HF. This bidirectional feedback system can lead to both acute and chronic functional impairments (14). Studies have shown that excessive activation of the sympathetic nerve in patients with heart failure (HF) may lead to myocardial remodeling, while abnormal processing of stress signals in the prefrontal cortex of patients with bipolar disorder (BD) may exacerbate their mood swings (15). Additionally, cytokines such as TNF-α and IL-6 play key roles in myocardial fibrosis in HF and neuroinflammation in mental disorders (16, 17). However, effective biomarkers and therapeutic strategies are currently lacking to address the complex pathology of such patients. Against this backdrop, this study aims to identify potential biomarkers associated with HF and BD through bioinformatics approaches, providing theoretical support for the development of more precise treatment regimens.

The precise mechanisms underlying the co-occurrence of BD and HF remain unclear, and key molecular factors linking the two conditions have yet to be thoroughly explored. Furthermore, the absence of comprehensive information regarding the risk factors for HF in patients with BD hampers the development of effective management strategies aimed at reducing mortality. To address this critical gap, our study aims to identify potential common potential diagnostic biomarkers for BD and HF through bioinformatics approaches, evaluate their diagnostic value, and predict potential therapeutic targets for these biomarkers, with the goal of uncovering novel treatment strategies for BD individuals with HF.

## 2 Materials and methods

### 2.1 Data acquisition

The GEOquery package was used to download the expression matrix data and GPL platform annotation files for HF and BD-related datasets from the GEO database (https://www.ncbi.nlm.nih.gov/geo/), and the expression matrix and sample metadata were extracted. The distinct function was used to remove duplicate genes, avoiding biases caused by gene repetition. Gene names were standardized to ensure consistency in gene identification. The expression values were log2 transformed to make the data conform more closely to the normal distribution assumption, while outliers (values less than or equal to 0) were handled for subsequent statistical analysis. The GSE57338 dataset included 136 normal heart tissue samples and 177 HF samples, while the GSE5389 dataset comprised 11 normal brain tissue samples and 10 BD samples. The GSE16499 dataset (15 ischemic heart failure samples and 15 age- and sex-matched control heart samples) and the GSE18312 dataset (9 BD samples and 8 controls samples) were used as validation sets.

### 2.2 Construction of co-expression networks

For the GSE57338 dataset, hierarchical clustering (complete linkage method) was employed to compute Euclidean distances between samples. Outliers were identified based on a cutting height (cutHeight = 110), and any identified outlier samples were removed. A co-expression network was then constructed using weighted gene co-expression network analysis (WGCNA) (2), selecting an appropriate soft threshold to ensure an R2 value exceeding 0.85 and connectivity tending to 0. Dynamic tree cutting was applied to classify genes into distinct modules. Pearson correlation was calculated between HF and the modules, with the modules showing the strongest positive and negative correlations selected as the key modules (P-value < 0.05). The genes within these modules were defined as key module genes (18, 19). The same approach was applied to identify key module genes related to BD.

### 2.3 Differential and enrichment analysis

Differentially expressed genes (DEGs) in the HF (GSE57338) and BD (GSE5389) datasets were identified using the ‘limma’ package (version 3.9) (20), applying thresholds of P < 0.05 and |log2Fold Change (FC)| > 0 (21). DEGs were visualized through volcano plots and heatmaps generated using the ‘ggplot2’ package (22). A Venn diagram was used to identify common DEGs (either upregulated or downregulated) between HF and BD. These common DEGs were further overlapped with BD-ModuleGenes and HF-ModuleGenes to pinpoint candidate genes. Enrichment analysis was performed on these candidate genes using the ‘clusterProfiler’ package (23), covering Gene Ontology (GO) and Kyoto Encyclopedia of Genes and Genomes (KEGG) pathways (P-value < 0.05).

### 2.4 Protein-protein interaction network and ROC analysis

To explore the protein-level interactions of candidate genes, a PPI network was constructed using the STRING database (https://STRING-db.org/) with a confidence score threshold of > 0.15 (24). The interaction types included weighted integration of experimental validation evidence, database inclusion evidence, predictive interaction evidence, and cross-species conservation evidence (25). The Degree algorithm within the CytoHubba plugin was used to calculate the Degree values of each gene in the network, with the top 10 genes ranked by Degree identified as hub genes. Diagnostic potential was assessed using the ‘pROC’ package (26), with genes that demonstrated diagnostic value (AUC > 0.7) and consistent expression patterns in the BD and HF training sets defined as potential diagnostic biomarkers. Additionally, further validation of biomarkers was conducted using the GSE16499 dataset related to HF and the GSE18312 dataset related to BD.

### 2.5 Gene set enrichment analysis

In the GSE57338 and GSE5389 datasets, disease samples were categorized into high- and low-expression groups based on the median expression levels of the potential diagnostic biomarkers. Differential expression analysis was then performed, and genes were ranked according to their log2FC values. To explore the potential KEGG pathways associated with the potential diagnostic biomarkers, GSEA was applied using the ‘clusterProfiler’ package (27), with an adjusted P-value threshold of < 0.05 for pathway selection.

### 2.6 Molecular network

To investigate the transcriptional regulation mechanisms of potential diagnostic biomarkers, the miRNet database was used to predict the transcription factors (TFs) and microRNAs (miRNAs) targeting these biomarkers. The ‘miRNA-mRNA-TF’ regulatory network was subsequently constructed using Cytoscape (version 3.9.1) software (28).

### 2.7 Potential drug prediction

Potential therapeutic drugs targeting the potential diagnostic biomarkers were identified through the DGIdb database (https://dgidb.org/). A gene-drug interaction network was then visualized using Cytoscape software.

### 2.8 Quantitative PCR

For experimental validation, total RNA was isolated from 10 pairs of frozen whole blood samples (10 HF samples *vs*. 10 control samples) using Trizol reagent (Ambion, Inc., A Thermo Fisher Scientific Company). cDNA synthesis was performed using the Reverse Transcription PrimeScript 1st Strand cDNA Synthesis Kit (Clontech Laboratories, Inc., A Takara Bio Company), and quantitative PCR was carried out with SYBR PremixExTaq™ (Clontech Laboratories, Inc., A Takara Bio Company). mRNA expression was measured using the CFX 96 system. The following primer sequences were employed for the PCR (**Table 1**).

**Table 1:** Primer information.

Primer	Sequence
FZD2	GCGAAGCCCTCATGAACAAG; TCCGTCCTCGGAGTGGTTCT.
EXT1	GAGGACGTGGGGTTTGACAT; CAAAAACCCCCTCTCCCCTC.
DCHS1	GAGTCTTTGCCACTGACCGA; TCAAGCACTGCAACATGCAC.
BMP4	ACTTCGAGGCGACACTTCTG; TCTGCTCTTCCTCCTCCTCC.
ALDH1A2	GCCTCTTCCTCTCTAACAGGC; GACGTCCCCTTTCTGAAGCA.
GAPDH	CGAAGGTGGAGTCAACGGATTT; ATGGGTGGAATCATATTGGAAC.

The PCR conditions were as follows: pre-denaturation at 95°C for 5 minutes, denaturation at 95°C for 15 seconds, annealing at 62°C for 30 seconds for 40 cycles, and final extension at 72°C for 30 seconds. qPCR data analysis was performed using the 2-ΔΔCt method.

### 2.9 Statistical analysis

All statistical analyses were conducted using R software (version 4.2.2) (R Core Team (2021). R: A language and environment for statistical computing. R Foundation for Statistical Computing, Vienna, Austria. https://www.R-project.org/). The Wilcoxon rank-sum test was used for comparing differences between the two groups in the bioinformatics analysis, and Pearson correlation was applied for correlation analysis. For RT-qPCR, the t-test was used to compare differences between groups. A P-value < 0.05 was considered statistically significant, and the significance threshold for the GSEA was set at an adjusted P-value < 0.05.

## 3 Results

### 3.1 Identification of differential and module genes for HF

**Figure 1:**
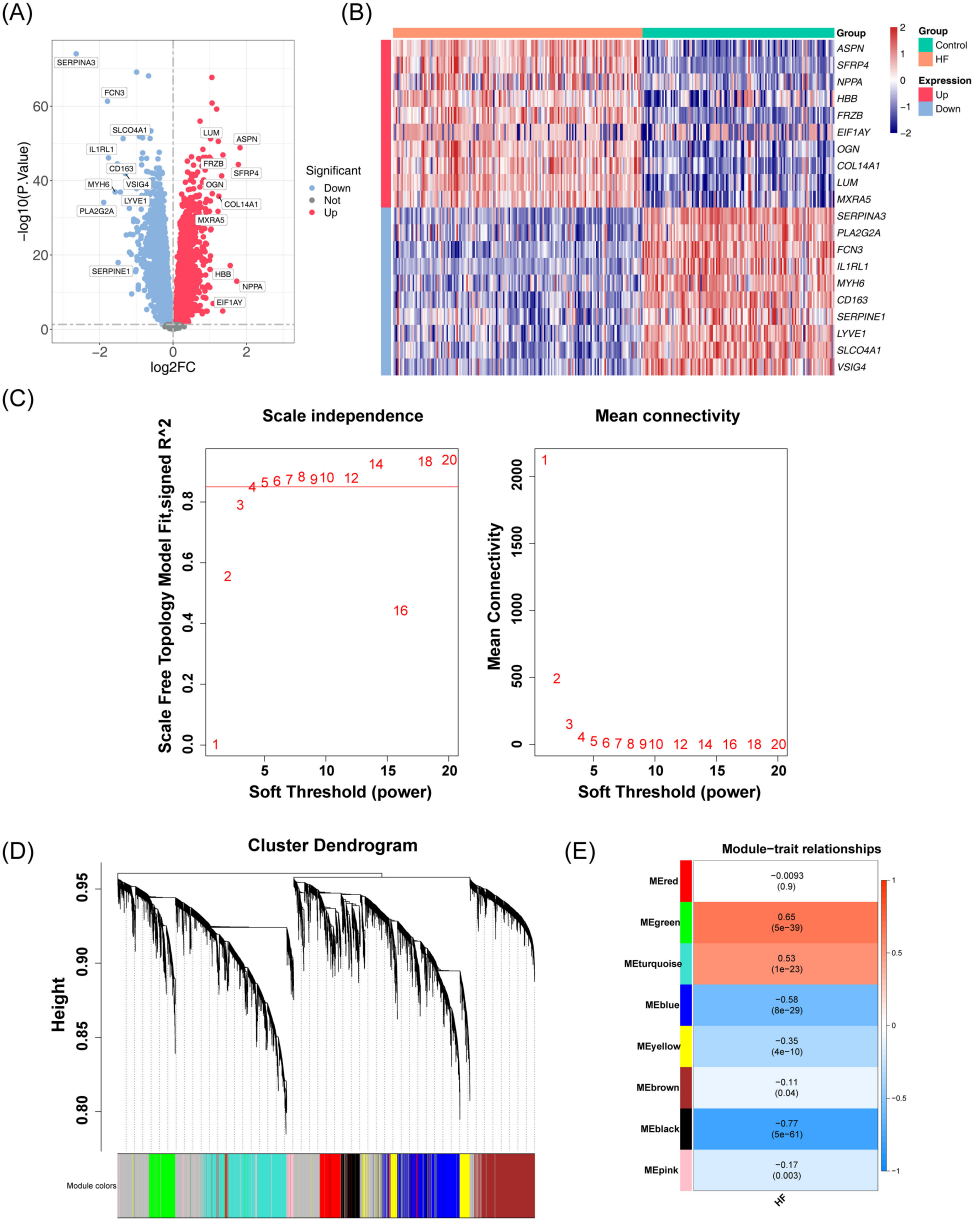
Analysis of DEGs and co-expression modules in the GSE57338 dataset. **(A)** Volcano plot displaying the distribution of DEGs in the GSE57338 dataset. A total of 11,665 DEGs were identified. Each point represents a gene: red indicates upregulated genes, blue indicates downregulated genes, and gray represents genes with no significant differential expression. **(B)** Heatmap illustrating the distribution of DEGs in the GSE57338 dataset. Gene expression levels are color-coded: red represents high expression, blue represents low expression, with the intensity of color reflecting the magnitude of gene expression. **(C)** Soft threshold screening. The scale-free fit index (left) and mean connectivity (right) are shown. A soft threshold of 5 was chosen for the network construction. **(D)** Hierarchical clustering tree of co-expression modules, with distinct colors representing different modules. A total of eight modules were identified. **(E)** Heatmap of module-trait correlations. Positive correlations are shown in red, while negative correlations are shown in blue. The horizontal axis represents traits, and the vertical axis represents the modules. Correlation coefficients are displayed in each grid, with larger absolute values indicating stronger correlations. Significance P-values are provided in parentheses, with smaller P-values indicating more statistically significant results.

A total of 11,665 DEGs were identified in the GSE57338 dataset for HF, including 6,347 downregulated and 5,318 upregulated genes (**Figures 1A, B**). Following clustering, two outlier samples were excluded (**Supplementary Figure S1**). A soft threshold of 5 was selected to construct the co-expression network (**Figure 1C**). The network was partitioned into eight distinct modules (**Figure 1D**), with the black and green modules showing significant associations with HF, making them key modules (**Figure 1E**). The number of genes in each module is shown in **Table 2**. A total of 1,691 genes were identified as key module genes related to HF.

**Table 2.** The number of genes in each module of GSE57338.

Modules	Number of genes	Significance
black	703	Significant
blue	2142	Significant
brown	2044	Significant
green	988	Significant
pink	655	Significant
turquoise	2683	Significant
yellow	1057	Significant
Red	970	Not significant

### 3.2 Identification of differential and module genes for BD

**Figure 2:**
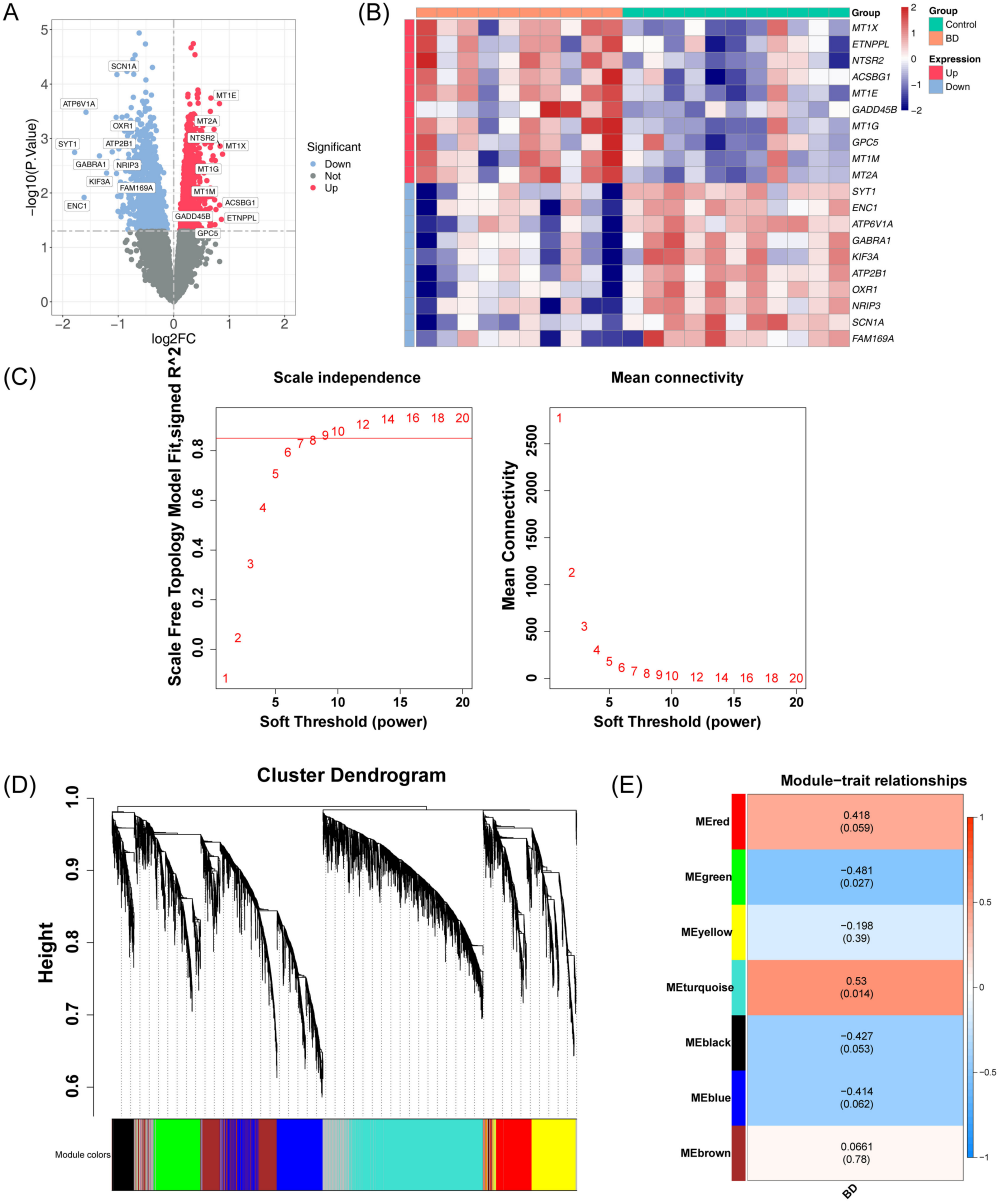
Integrated analysis of DEGs and co-expression modules in the GSE5389 dataset. **(A)** Volcano plot illustrating the distribution of DEGs in the GSE5389 dataset. A total of 2,549 DEGs were identified. Each point represents a gene: red indicates upregulated genes, blue indicates downregulated genes, and gray represents genes with no significant differential expression. **(B)** Heatmap showing the distribution of DEGs in the GSE5389 dataset. The intensity of the color represents the gene expression level, with red indicating high expression and blue indicating low expression. **(C)** Soft threshold screening. The scale-free fit index (left) and mean connectivity (right) are shown. A soft threshold of 9 was selected for the network construction. **(D)** Hierarchical clustering tree of co-expression modules, with distinct colors representing different modules. Seven modules were identified. **(E)** Heatmap of module-trait correlations. Positive correlations are depicted in red, while negative correlations are shown in blue. The horizontal axis represents traits, and the vertical axis represents the modules. Each grid shows the correlation coefficient values, with larger absolute values indicating stronger correlations. The significance P-values are displayed in parentheses, with smaller P-values indicating more statistically significant results.

In the GSE5389 dataset for BD, 2,549 DEGs were identified, including 1,073 downregulated and 1,476 upregulated genes (**Figures 2A, B**). No outlier samples were detected after clustering the data (**Supplementary Figure S2**). A soft threshold of 9 was selected for constructing the co-expression network (**Figure 2C**), resulting in the identification of seven modules (**Figure 2D**). The number of genes in each module is shown in **Table 3**. Among these, the turquoise and green modules showed significant associations with BD (**Figure 2E**), with 3,935 genes identified as key module genes associated with BD.

**Table 3:** The number of genes in each module of GSE5389.

Modules	Number of genes	Significance
green	1001	Significant
turquoise	2934	Significant
black	499	Not significant
blue	1539	Not significant
brown	1218	Not significant
grey	932	Not significant
red	880	Not significant

### 3.3 Biomarkers screening in HF and BD

**Figure 3:**
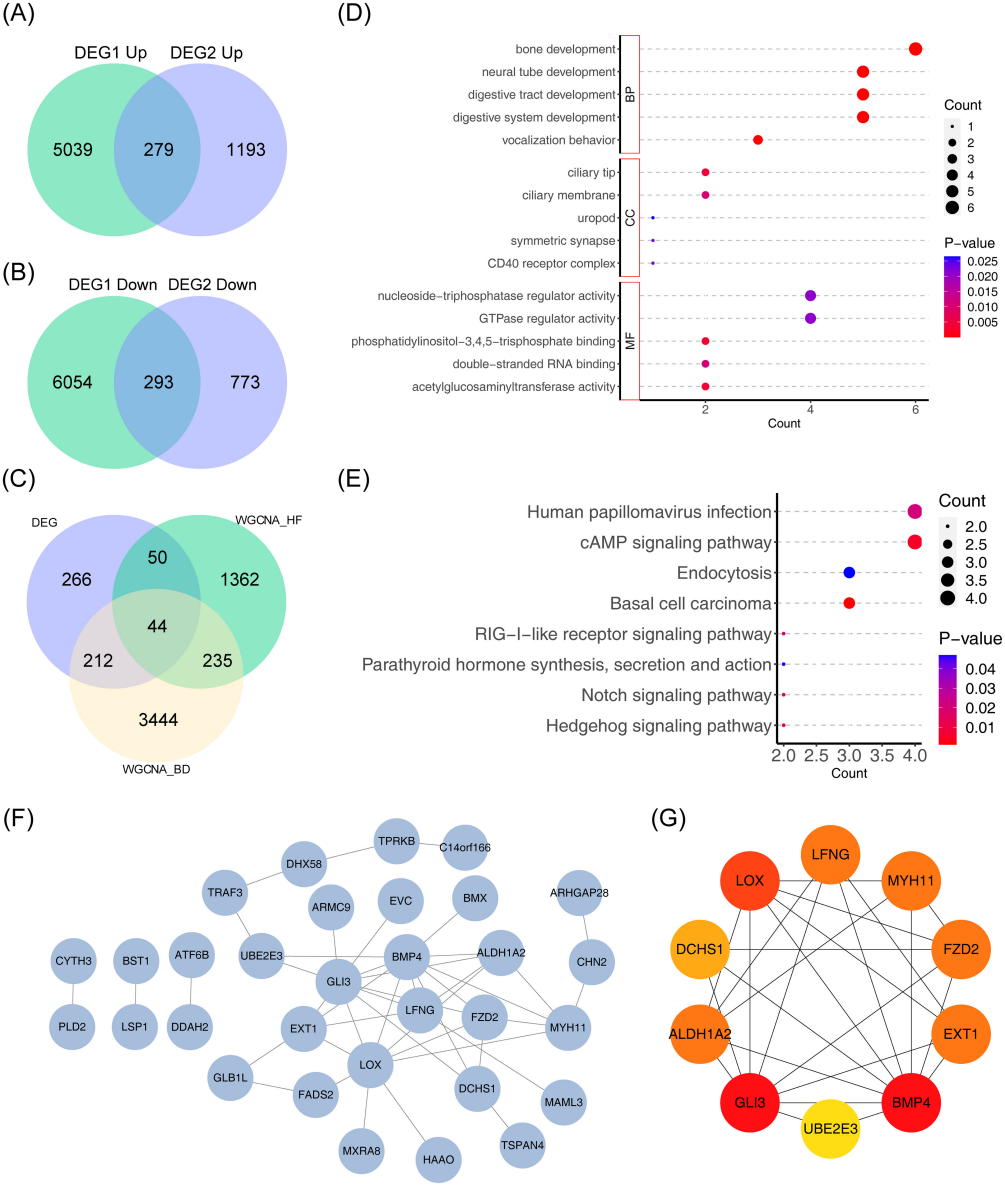
Genomic analysis visualizations: Venn diagrams, intersection analysis, enrichment plots, and protein network. **(A)** Venn diagram depicting the intersection of differentially upregulated genes across the two datasets. **(B)** Venn diagram depicting the intersection of differentially downregulated genes across the two datasets. **(C)** Venn diagram for gene intersections of shared variance modules. **(D, E)** Bubble plots of GO and KEGG enrichment analysis. The vertical axis represents pathway names, while the horizontal axis indicates the number of genes enriched in each pathway. Larger bubbles correspond to a greater gene count. A color gradient from blue to red reflects increasing significance. **(F)** Protein interaction network diagram. **(G)** Degree-based top 10 gene interaction network, with color changes from yellow to red denoting increasing Degree values.

A total of 572 common DEGs were identified through the intersection of DEGs in HF and BD, comprising 279 upregulated genes (**Figure 3A**) and 293 downregulated genes (**Figure 3B**). Additionally, 572 common DEGs, 1,691 module genes strongly associated with HF in the GSE57338 dataset, and 3,967 module genes associated with BD in the GSE5389 dataset were intersected, resulting in 44 candidate genes (**Figure 3C**). To explore the potential mechanisms of the 44 candidate genes, functional enrichment analysis was performed. The top five GO terms indicated a predominant association with ‘neural tube development’ (**Figure 3D**). The top eight KEGG pathways highlighted strong involvement in the ‘RIG-I-like receptor signaling pathway’ and the ‘cAMP signaling pathway’ (**Figure 3E**). Furthermore, a PPI network encompassing 31 nodes and 44 edges was constructed for the candidate genes (**Figure 3F**). Ten hub genes (*UBE2E3, FZD2, GLI3, EXT1, DCHS1, MYH11, BMP4, LOX, LFNG*, and *ALDH1A2*) were identified using the Degree algorithm (**Figure 3G**).

### 3.4 Potential diagnostic biomarkers screening in HF and BD

**Figure 4:**
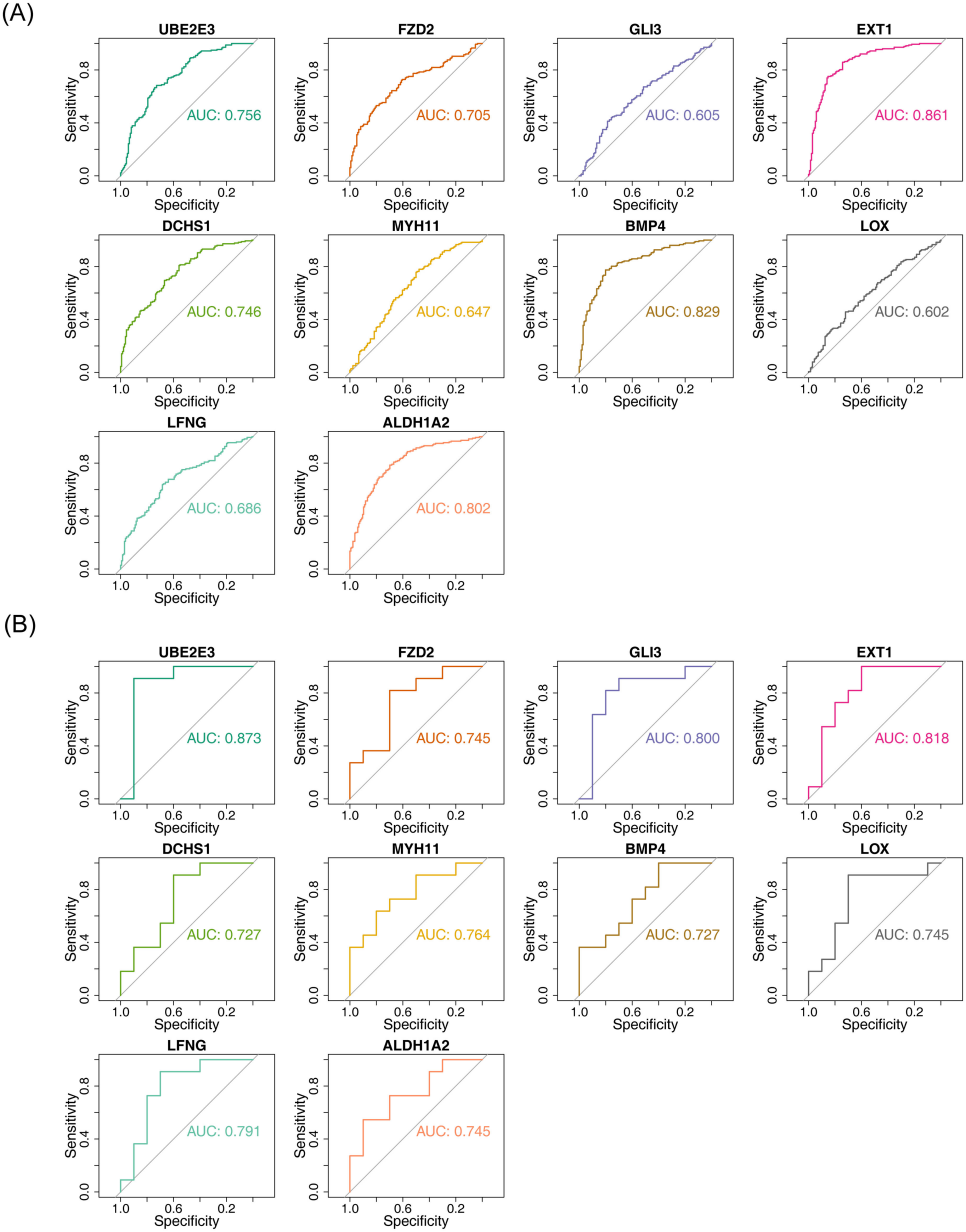
ROC analysis of hub genes (GSE57338 vs GSE5389). **(A)** ROC analysis for hub genes in GSE57338, where an AUC greater than 0.7 indicates relatively high diagnostic accuracy for HF. **(B)** ROC analysis for hub genes in GSE5389, with an AUC greater than 0.7 suggesting high diagnostic accuracy for BD.

**Figure 5:**
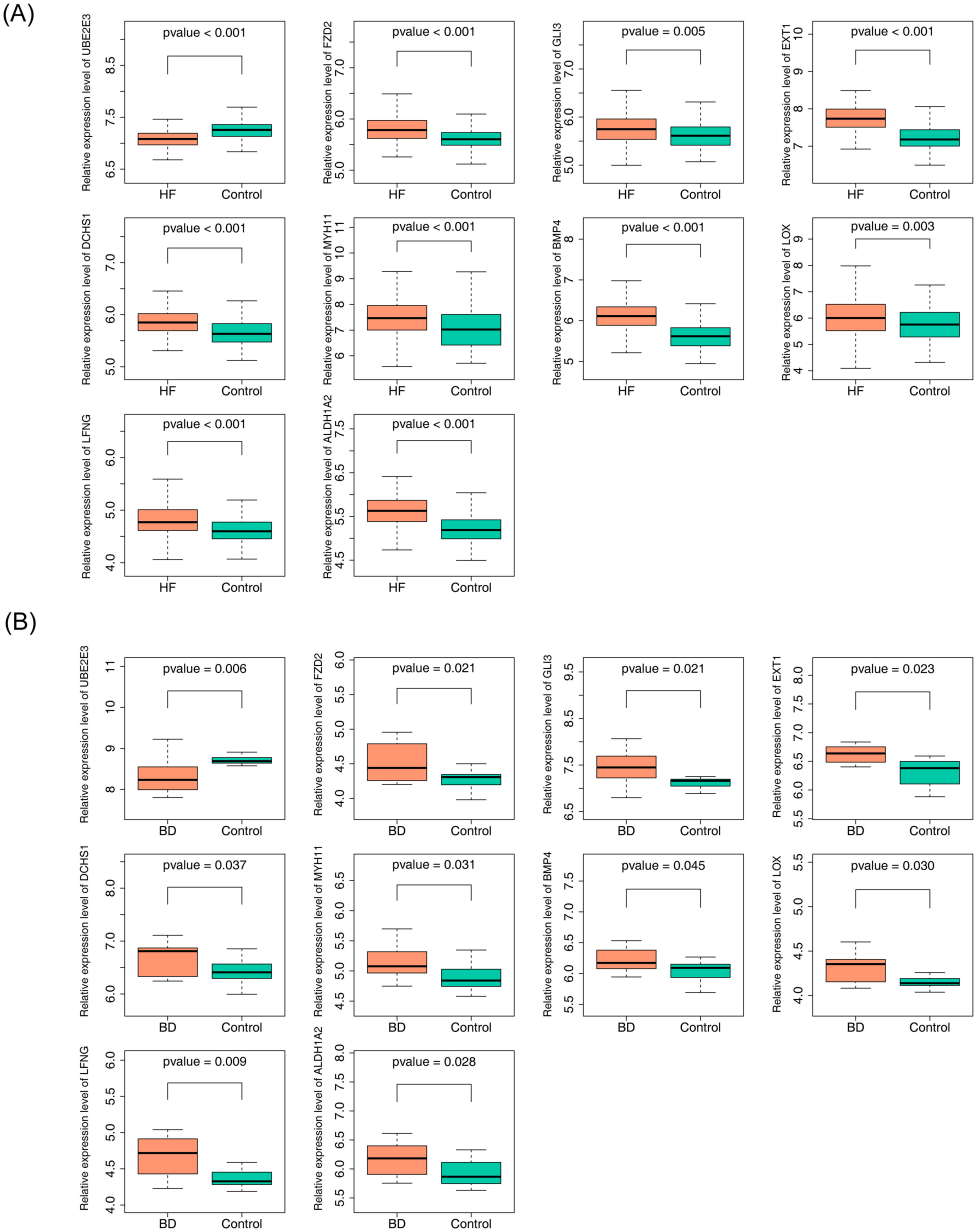
Expression analysis of hub genes (GSE57338 vs GSE5389). **(A)** Expression analysis of hub genes in GSE57338. **(B)** Expression analysis of hub genes in GSE5389.

The diagnostic accuracy of the hub genes for HF and BD was assessed using ROC curves (**Figures 4A, B**). The analysis revealed that six genes (*UBE2E3, FZD2, EXT1, DCHS1, BMP4*, and *ALDH1A2*) exhibited strong diagnostic performance for both HF and BD (AUC > 0.7). Additionally, increased expression of *FZD2, EXT1, DCHS1, BMP4*, and *ALDH1A2* was observed in the disease group (HF and BD), whereas *UBE2E3* showed low expression (**Figures 5A, B**). Consequently, these six genes were defined as potential diagnostic biomarkers for HF and BD. In the validation set GSE16499, the diagnostic performance of UBE2E3, EXT1, DCHS1, BMP4, and ALDH1A2 was relatively good (AUC > 0.6), while the diagnostic performance of FZD2 was relatively low (**Supplementary Figure 1**). In the validation set GSE18312, UBE2E3, EXT1, DCHS1, and FZD2 demonstrated relatively good diagnostic performance (AUC > 0.6), while the diagnostic performance of BMP4 and ALDH1A2 was relatively low (**Supplementary Figure 2**).

### 3.5 GSEA of potential diagnostic biomarkers

**Figure 6:**
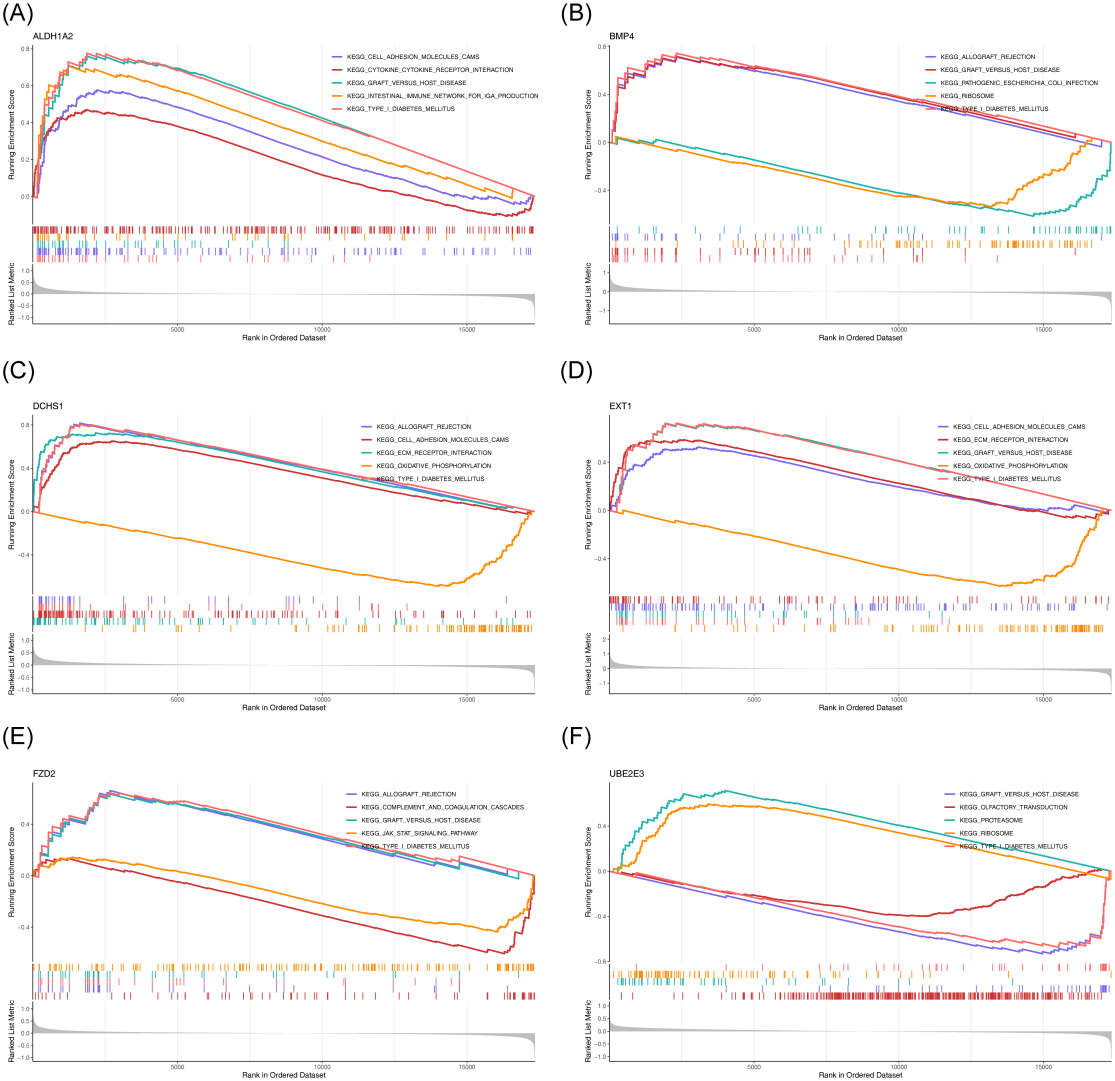
GSEA results for ALDH1A2 **(A)**, BMP4 **(B)**, DCHS1 **(C)**, EXT1 **(D)**, FZD2 **(E)**, and UBE2E3 **(F)** in GSE57338. Each sub-Figure is composed of three components: the top section displays an enrichment score line graph, with each line representing a distinct pathway. The second section highlights the genes within the gene set using lines, while the third section illustrates the distribution of rank values for all genes.

**Figure 7:**
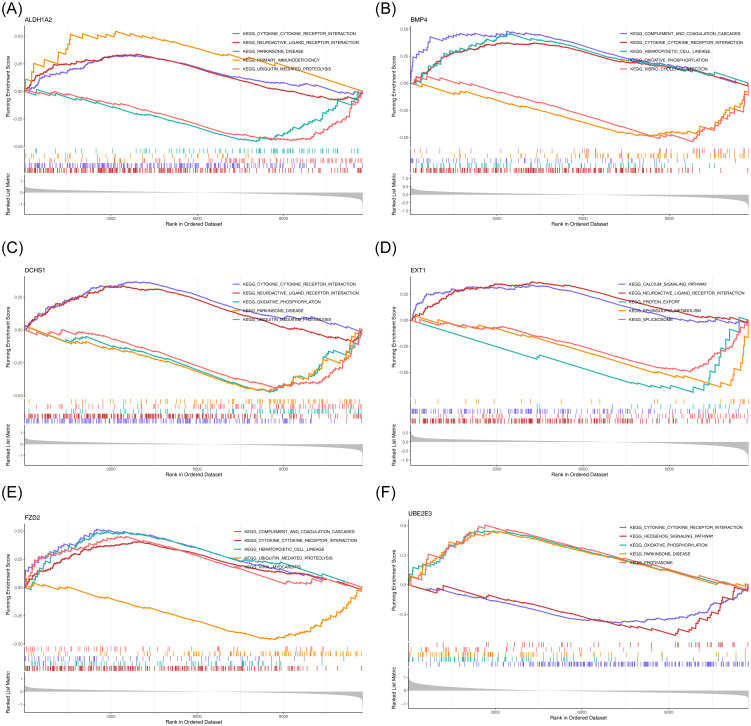
GSEA results for ALDH1A2 **(A)**, BMP4 **(B)**, DCHS1 **(C)**, EXT1 **(D)**, FZD2 **(E)**, and UBE2E3 **(F)** in GSE5389.

To explore the potential roles of the six potential diagnostic biomarkers, single-gene GSEA was performed *ALDH1A2* was primarily associated with ‘cytokine-cytokine receptor interaction’ in HF and ‘ubiquitin-mediated proteolysis’ in BD (**Figures 6A**, **7A**). *BMP4* was predominantly involved in ‘ribosome’ and ‘cytokine-cytokine receptor interaction’ pathways in HF and BD, respectively (**Figures 6B**, **7B**). *DCHS1* was chiefly linked to ‘cytokine-cytokine receptor interaction’ in BD and ‘ECM receptor interaction’ in HF (**Figures 6C**, **7C**). *EXT1* was enriched in ‘ECM receptor interaction’ in HF and ‘calcium signaling pathway’ in BD (**Figures 6D**, **7D**). *FZD2* was primarily associated with the ‘JAK-STAT signaling pathway’ in HF and ‘cytokine-cytokine receptor interaction’ in BD (**Figures 6E**, **7E**). *UBE2E3* was primarily enriched in the ‘ribosome’ pathway in HF and the ‘proteasome’ pathway in BD (**Figures 6F**, **7F**).

### 3.6 Analysis of regulatory relationships

**Figure 8:**
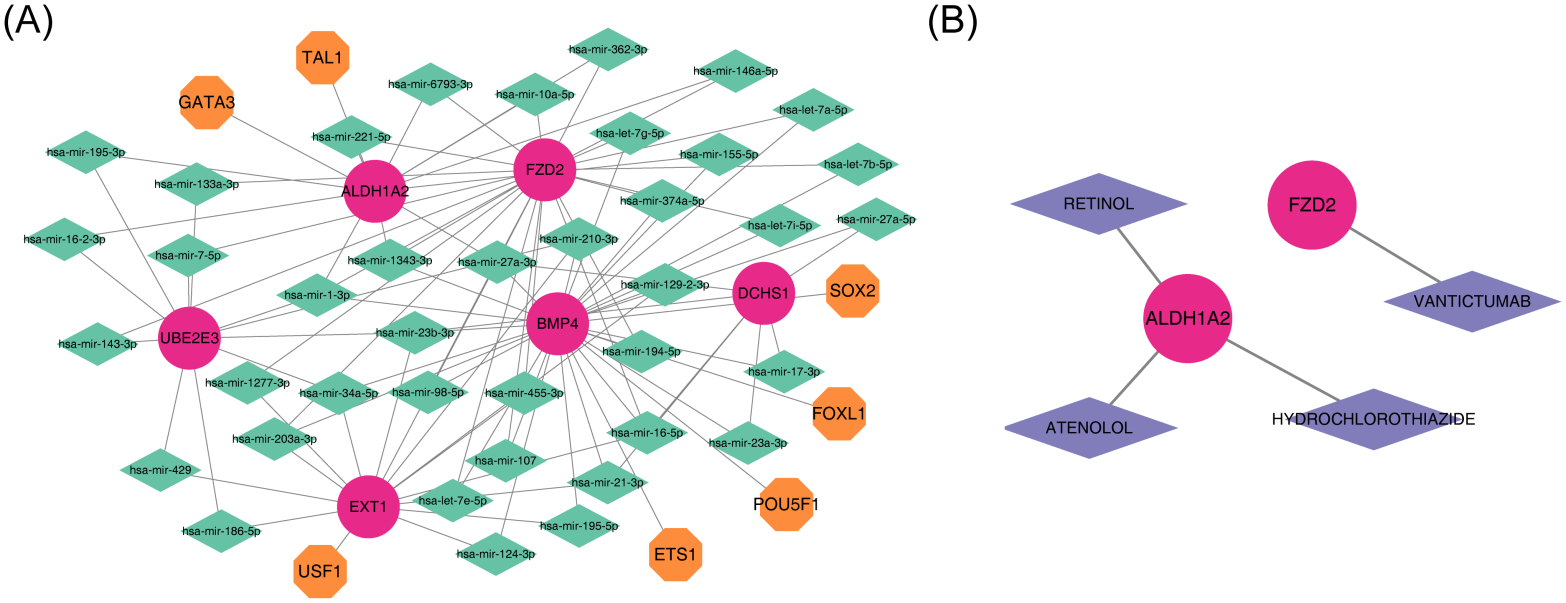
Integrated regulatory network and gene-drug interaction in diagnostics. **(A)** Diagram of the miRNA-mRNA-TF regulatory network, with red representing diagnostic markers, orange indicating TFs, and green representing miRNAs. **(B)** Diagnostic gene-drug interaction network, with red for diagnostic markers and purple for drugs.

To investigate the regulatory mechanisms of the potential diagnostic biomarkers, a ‘miRNA-mRNA-TF’ network was constructed (**Figure 8A**), comprising 52 nodes and 199 edges. Notably, hsa-miR-1343-3p was linked to *ALDH1A2, BMP4*, and *FZD2.* Furthermore, four drugs—VANTICTUMAB, RETINOL, HYDROCHLOROTHIAZIDE, and ATENOLOL—were identified as potential therapeutics for *ALDH1A2* and *FZD2* (**Figure 8B**; **Supplementary Table 1**).

### 3.7 Expression levels of *FZD2, EXT1, DCHS1, BMP4*, and *ALDH1A2*

**Figure 9:**
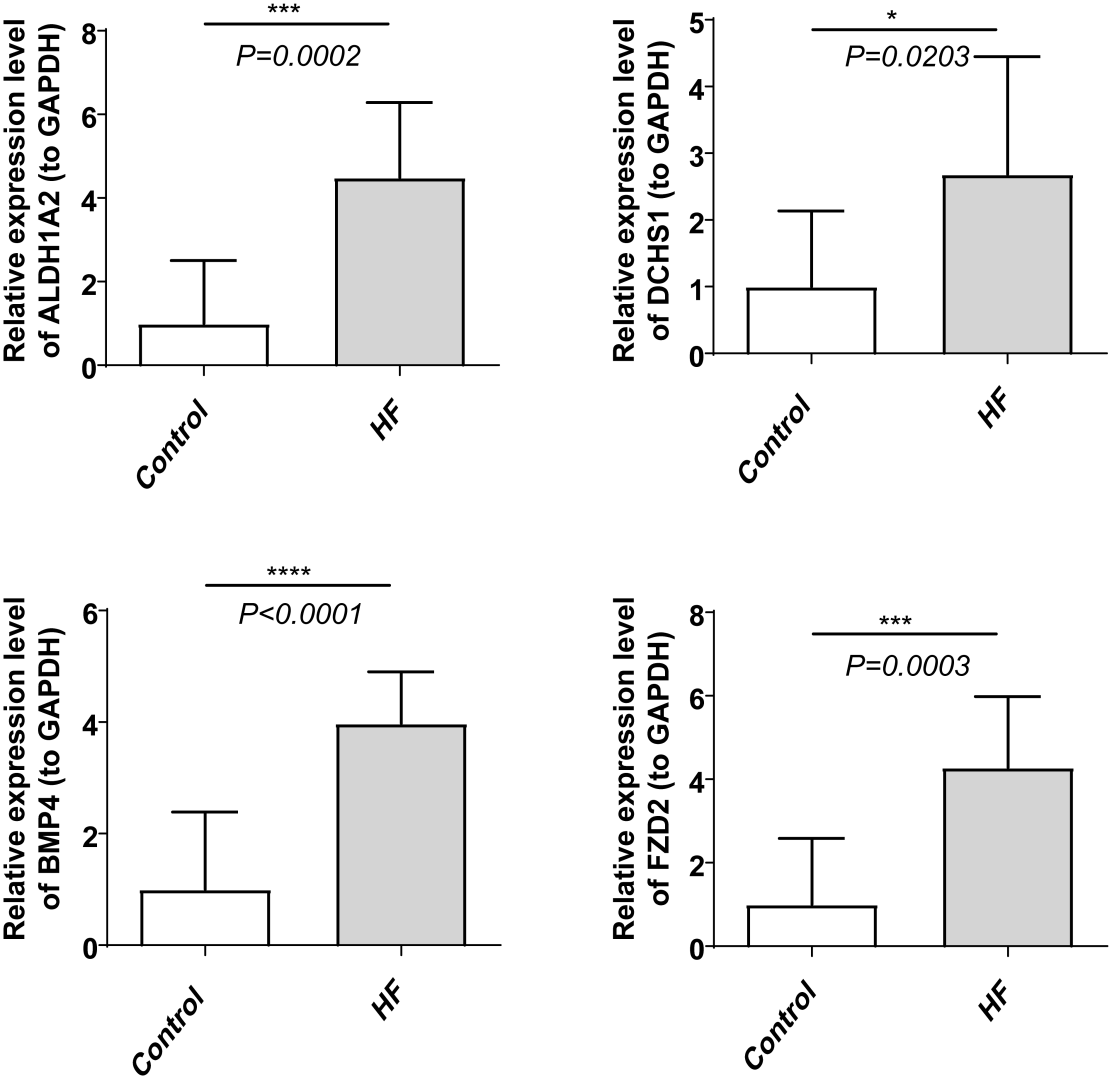
Expression patterns of FZD2, DCHS1, BMP4, and ALDH1A2 in HF and control groups. ****indicates P < 0.0001, ***indicates P < 0.001, and *indicates P < 0.05.

qPCR results validated that the expression patterns of *FZD2, EXT1, DCHS1, BMP4*, and *ALDH1A2* were consistent with the dataset observations. In comparison to healthy controls, *FZD2, DCHS1, BMP4*, and *ALDH1A2* were significantly upregulated in HF samples (P < 0.05, **Figure 9**).

## 4 Discussion

Research showed that patients with BD have a life expectancy of 8–12 years shorter than healthy individuals, possibly due to a higher prevalence of diabetes, metabolic syndrome, and CVD (1, 11, 29). CVD represents a significant mortality risk factor in manic BD, with patients with BD suffering from CVD having an 8-fold higher mortality rate than healthy individuals under 40 years of age (30). Previous studies have identified common risk factors for CVD mortality in patients with mental illness, including smoking, poor diet, inflammatory factors, and psychotropic drug use (31). Despite being a prevalent CVD, the relationship between HF and BD remains unclear. This study used bioinformatics methods to analyze potential common diagnostic biomarkers between BD and HF, aiming to provide a new theoretical basis for future research on the common biological mechanism of HF and BD.

A joint analysis of two datasets from the GEO database was conducted to identify common DEGs in BD and HF. This analysis, combined with WGCNA, allowed for the identification of module genes associated with each disease. A total of 44 common differential module genes were uncovered. These genes were subjected to enrichment analysis, and a PPI network was constructed using the STRING database to identify hub genes, resulting in the identification of 10 hub genes. ROC analysis of these hub genes led to the identification of six genes (*UBE2E3, FZD2, EXT1, DCHS1, BMP4*, and *ALDH1A2*) with diagnostic potential. Finally, qPCR validation of five upregulated genes (*FZD2, EXT1, DCHS1, BMP4*, and *ALDH1A2*) in HF blood samples confirmed that the expression trends of *FZD2, DCHS1, BMP4*, and *ALDH1A2* were consistent with those observed in the GEO database.

Dachsous cadherin-related 1 (*DCHS1*), a gene involved in tissue development and organization, encodes a calcium-dependent cell-adhesion protein. *DCHS1* plays critical roles in regulating the proliferation and differentiation of neuroprogenitor cells. It is also essential for proper mitral valve morphogenesis in the heart, regulating cell migration during valve formation (6, 32). Whole-exome sequencing has identified 122 BD-related genes, including *DCHS1* (33). Moreover, abnormal neurodevelopment is considered a potential cause of BD (34), in which *DCHS1* plays a pivotal role. As a key gene in cerebral cortex development, alterations in *DCHS1* expression or function can lead to abnormalities in neuronal migration, differentiation, and synaptic connections, increasing the risk of BD (35). Additionally, *DCHS1* is involved in regulating the Hippo signaling pathway (36), which is crucial in cell proliferation, apoptosis, and differentiation. Dysregulation of this pathway can impact neuron survival and function, contributing to the onset and progression of mental illnesses (37, 38). In our dataset, *DCHS1* expression was upregulated in both HF and BD, suggesting its potential as a target gene for further study in BD individuals with concurrent HF.

Bone morphogenetic protein 4 (*BMP4*), a member of the TGF-beta superfamily, acts as a growth factor involved in several biological processes, including vascular development and angiogenesis (39, 40). Numerous studies have highlighted the critical role of BMP4 in the pathogenesis of HF, identifying it as a key therapeutic target for intervention (41–43). In our dataset, *BMP4* expression was upregulated in both HF and BD, which aligns with findings from Wu et al. (43), who observed elevated levels of *BMP4* precursor protein in mouse hearts 24 hours after infarction. Their study further demonstrated that recombinant *BMP4* had protective effects on cultured cardiomyocytes. Additionally, Wen et al. showed that *BMP4* mediates various aspects of pathological cardiac hypertrophy, including cardiac hypertrophy, apoptosis, fibrosis, and ion channel remodeling (41). *BMP4* also downregulates the activation of naive CD4+ T cells and inhibits IFN-γ production by these cells, without increasing regulatory T cell numbers. Furthermore, BMP4 can influence T cell glycolysis and Hif1α expression (44), suggesting that *BMP4* may inhibit IFN-γ production by CD4+ T cells *in vivo*, potentially affecting immune responses and contributing to BD development. However, no reports have yet linked BMP4 to BD directly.

Frizzled-2 (*FZD2*) functions as a receptor for Wnt proteins, with most frizzled receptors associated with the canonical beta-catenin signaling pathway. This pathway involves the activation of disheveled proteins, inhibition of GSK-3 kinase, nuclear accumulation of beta-catenin, and subsequent activation of Wnt target genes (45). Research suggests that *FZD* family members may act as predisposition genes for schizophrenia (46, 47). Additionally, studies indicate that *FZD2* prevents adult mouse cardiomyocytes from re-entering the cell cycle by inhibiting Yes-associated protein (YAP), thus protecting the myocardium after myocardial infarction by preventing excessive cardiomyocyte proliferation and fibrosis. As a receptor for Wnt, *FZD2* may also influence neurodevelopment via the Wnt/β-catenin pathway (48), suggesting its potential role in the development of BD. Further investigation of *FZD2*’s mechanism in BD pathogenesis is warranted. Aldehyde Dehydrogenase 1, Family Member A2 (*ALDH1A2*), which encodes retinal dehydrogenase 2, plays a pivotal role in synthesizing retinoic acid from vitamin A during early development and is strongly associated with heart disease (49, 50). *ALDH1A2* is vital for cardiac development. Regulating its expression can impact cardiac lesions, particularly in the context of chronic inflammation and fibrosis in HF (51). Additionally, *ALDH1A2* is involved in retinoic acid synthesis, a critical component of the retinoic acid signaling pathway, which is essential for neurodevelopment (49, 51). Dysregulation of *ALDH1A2* may result in abnormal retinoic acid levels, which in turn can affect the development, differentiation, and function of neurons, thereby increasing the risk of developing BD. However, no studies have yet explored the role of *ALDH1A2* in BD.

GSEA results indicated that genes such as *ALDH1A2, DCHS1*, and *EXT1* were significantly enriched in pathways including cytokine-cytokine receptor interaction and ECM-receptor interaction. The Cytokine-Cytokine Receptor Interaction pathway plays a pivotal role in the progression of HF. On one hand, this pathway promotes the over-activation of pro-inflammatory cytokines, such as tumor necrosis factor-alpha (TNF-α), exacerbating the inflammatory response, inducing cardiomyocyte damage and apoptosis, reducing myocardial contractility, and accelerating the deterioration of HF (52). On the other hand, interleukin 6 (IL-6) triggers downstream signaling pathways that lead to myocardial remodeling, altering the heart’s structure and decreasing its pumping function (53). The ECM-receptor interaction pathway is pivotal in the pathogenesis of both HF and BD, influencing each disease through different mechanisms. In the context of HF, an imbalance in ECM-receptor interactions disrupts the synthesis and degradation of extracellular matrix proteins, such as collagen (54, 55). Excessive ECM deposition increases myocardial stiffness, impairing diastolic function and affecting the heart’s filling capacity. Additionally, abnormal activation of ECM receptors, such as integrins, promotes the activation and proliferation of cardiac fibroblasts through downstream signaling pathways, accelerating myocardial fibrosis and further reducing the heart’s compliance and contractile function (56, 57). In BD, ECM-receptor interactions in the brain are also crucial. ECM receptors on neurons and glial cells modulate the plasticity of nerve synapses by regulating intercellular signaling (58, 59). Dysregulation of ECM receptor signaling may alter neurotransmitter transmission and disrupt neuronal connections, contributing to emotional regulation disorders, increasing the risk of BD, or influencing its progression (60, 61). The involvement of these pathways in both HF and BD offers valuable insights into the comorbidity mechanisms of these diseases and presents potential targets for future therapeutic interventions.

Analysis of the regulatory network revealed that hsa-mir-1343-3p simultaneously targets *ALDH1A2, BMP4*, and *FZD2*. hsa-mir-1343-3p is a miRNA, a class of small non-coding RNA molecules that regulate gene expression by binding to target mRNAs. These molecules play pivotal roles in various physiological and pathological cellular processes (62). hsa-mir-1343-3p inhibits autophagy by targeting *ATG7* (63), a critical process for maintaining cardiomyocyte health, which is linked to the onset and progression of HF (64). Consequently, the regulation of autophagy by hsa-mir-1343-3p may influence cardiomyocyte survival and function, thereby modulating HF progression. Furthermore, hsa-mir-1343-3p may regulate dopamine synthases, transporters, and receptors, affecting the development of BD (65–67). In conclusion, as a potential key regulatory molecule, hsa-mir-1343-3p targets multiple critical genes and modulates autophagy- and dopamine-related processes, may playing a significant role in the pathogenesis of both HF and BD and offering valuable insights for the exploration of these diseases’ mechanisms and the development of novel therapeutic strategies.

This study identified four drugs with potential therapeutic effects on *ALDH1A2* and *FZD2*, including VANTICTUMAB, RETINOL, HYDROCHLOROTHIAZIDE, and ATENOLOL, which may prove beneficial for treating BD individuals with HF. The study results showed that patients treated with ATENOLOL demonstrated significant improvements in aggravated heart failure and death events (68). Another study indicated that a combination pill containing ATENOLOL and HYDROCHLOROTHIAZIDE significantly reduced low-density lipoprotein cholesterol and systolic blood pressure, with a lower incidence of cardiovascular events compared to the placebo group, effectively decreasing the incidence of cardiovascular events in individuals with higher cardiovascular risk (69). Studies have shown that when hydrochlorothiazide is used in combination with Dapagliflozin, it can synergistically improve hemodynamics and ejection fraction in early intervention, and reduce plasma B-type natriuretic peptide concentration. Moreover, hydrochlorothiazide enhances the inhibitory effect of Dapagliflozin on NHE activity by inhibiting the expression of NHE1, thereby further improving cardiac function (70). These medications may improve the cardiovascular status of BD patients by stabilizing their blood pressure. This finding provides new therapeutic insights for BD patients with comorbid hypertension and HF. However, considering the potential and limitations of these medications in clinical application, their efficacy in BD patients with comorbid HF cannot be fully proven. Although these medications may affect relevant disease pathways by acting on diagnostic genes, their actual efficacy in BD and HF still needs to be validated by further experimental and clinical studies.

The six diagnostic genes identified in this study hold significant potential and can serve as a foundation for future research. To date, no conjoint analysis of BD and HF has been reported. By leveraging public databases and bioinformatics methods, this study preliminarily explored the shared pathogenesis of BD and HF, revealing a potential common underlying mechanism and offering new opportunities for diagnosing and treating patients affected by both conditions. However, the study still has some limitations. First, relying on existing databases and the small sample sizes of BD datasets and qPCR validation may increase the risk of overfitting and false-positive module detection, which may affect the generalizability and accuracy of the results. Second, although some findings were validated by qPCR, we recognize that the BD dataset was derived from brain tissue and the HF dataset from heart tissue, and current validation was only performed in HF samples. Tissue differences may affect the universality of the results. In future studies, we plan to seek samples from individuals with both BD and HF for more comprehensive analysis. Additionally, as this study is still in the preliminary exploration stage, to capture more potential biological differences, we used |log2FC| > 0 and uncorrected P-values as thresholds for screening DEGs, as well as a slightly lower STRING confidence threshold, which may include some biologically irrelevant changes. In the future, we will combine stricter threshold criteria to optimize the analysis process and ensure that the screened genes are more consistent with the actual biological context. Finally, future research will expand the sample size and introduce more brain-derived data, including brain specimens or autopsy samples from BD patients, to further confirm the performance of these genes in the brain. Meanwhile, CRISPR-Cas9 technology will be used to knockout or overexpress these genes, and through cell proliferation, apoptosis, and metabolism experiments, the effects of these genes on myocardial cells and nerve cells will be evaluated to further clarify their roles in HF and BD. In addition, more experiments will be needed in the future to verify the specific common mechanisms between BD and HF.

## Data Availability

The datasets presented in this study can be found in online repositories. The names of the repository/repositories and accession number(s) can be found in the article/**Supplementary Material**.

## References

[1] Chen PH Tsai SY Pan CH Chang HM Chen YL Su SS. Incidence and risk factors of sudden cardiac death in bipolar disorder across the lifespan. J Affect Disord. (2020) 274:210–7. doi: 10.1016/j.jad.2020.05.094, PMID: 32469806

[2] Langfelder P Horvath S. WGCNA: an R package for weighted correlation network analysis. BMC Bioinf. (2008) 9:559. doi: 10.1186/1471-2105-9-559, PMID: 19114008 PMC2631488

[3] Stahl EA Breen G Forstner AJ McQuillin A Ripke S Trubetskoy V. Genome-wide association study identifies 30 loci associated with bipolar disorder. Nat Genet. (2019) 51:793–803. doi: 10.1038/s41588-019-0397-8, PMID: 31043756 PMC6956732

[4] Wartchow KM Scaini G Quevedo J. Glial-neuronal interaction in synapses: A possible mechanism of the pathophysiology of bipolar disorder. Adv Exp Med Biol. (2023) 1411:191–208. doi: 10.1007/978-981-19-7376-5_9, PMID: 36949311

[5] Xu E Nguyen L Hu R Stavish CM Leibenluft E Linke JO. The uncinate fasciculus in individuals with and at risk for bipolar disorder: A meta-analysis. J Affect Disord. (2022) 297:208–16. doi: 10.1016/j.jad.2021.10.045, PMID: 34699854 PMC8631233

[6] Wolfers T Doan NT Kaufmann T Alnaes D Moberget T Agartz I. Mapping the heterogeneous phenotype of schizophrenia and bipolar disorder using normative models. JAMA Psychiatry. (2018) 75:1146–55. doi: 10.1001/jamapsychiatry.2018.2467, PMID: 30304337 PMC6248110

[7] Mohan IK Baba K Iyyapu R Thirumalasetty S Satish OS. Advances in congestive heart failure biomarkers. Adv Clin Chem. (2023) 112:205–48. doi: 10.1016/bs.acc.2022.09.005, PMID: 36642484

[8] Vrachatis DA Papathanasiou KA Giotaki SG Raisakis K Kaoukis A Kossyvakis C. Advances in the management of heart failure with reduced ejection fraction; the role of SGLT2is, ARNI, myotropes, vericiguat, and anti-inflammatory agents: A mini-review. Curr Pharm Des. (2023) 29:509–18. doi: 10.2174/1381612829666230316142450, PMID: 36927423

[9] Gladigau EL Fazio TN Hannam JP Dawson LM Jones SG. Increased cardiovascular risk in patients with severe mental illness. Intern Med J. (2014) 44:65–9. doi: 10.1111/imj.12319, PMID: 24383746

[10] Nielsen RE Banner J Jensen SE. Cardiovascular disease in patients with severe mental illness. Nat Rev Cardiol. (2021) 18:136–45. doi: 10.1038/s41569-020-00463-7, PMID: 33128044

[11] Chen PH Chiang SJ Hsiao CY Shen RS Lin YK Chung KH. Echocardiographic study of cardiac structure and function in people with bipolar disorder after midlife. J Affect Disord. (2022) 296:428–33. doi: 10.1016/j.jad.2021.09.089, PMID: 34606806

[12] Ortiz A Bradler K Moorti P MacLean S Husain MI Sanches M. Reduced heart rate variability is associated with higher illness burden in bipolar disorder. J Psychosom Res. (2021) 145:110478. doi: 10.1016/j.jpsychores.2021.110478, PMID: 33820643

[13] Ski CF Taylor RS McGuigan K Long L Lambert JD Richards SH. Psychological interventions for depression and anxiety in patients with coronary heart disease, heart failure or atrial fibrillation. Cochrane Database Syst Rev. (2024) 4:CD013508. doi: 10.1002/14651858.CD013508.pub3, PMID: 38577875 PMC10996021

[14] Doehner W Celutkiene J Yilmaz MB Coats AJS. Heart failure and the heart-brain axis. QJM. (2023) 116:897–902. doi: 10.1093/qjmed/hcad179, PMID: 37481714

[15] Díaz HS Toledo C Andrade DC Marcus NJ Del Rio R. Neuroinflammation in heart failure: new insights for an old disease. J Physiol. (2020) 598:33–59. doi: 10.1113/JP278864, PMID: 31671478

[16] Dihoum A Brown AJ McCrimmon RJ Lang CC Mordi IR. Dapagliflozin, inflammation and left ventricular remodelling in patients with type 2 diabetes and left ventricular hypertrophy. BMC Cardiovasc Disord. (2024) 24:356. doi: 10.1186/s12872-024-04022-7, PMID: 38997620 PMC11241903

[17] Miller AH Maletic V Raison CL. Inflammation and its discontents: the role of cytokines in the pathophysiology of major depression. Biol Psychiatry. (2009) 65:732–41. doi: 10.1016/j.biopsych.2008.11.029, PMID: 19150053 PMC2680424

[18] Lv JH Hou AJ Zhang SH Dong JJ Kuang HX Yang L. WGCNA combined with machine learning to find potential biomarkers of liver cancer. Med (Baltimore). (2023) 102:e36536. doi: 10.1097/MD.0000000000036536, PMID: 38115320 PMC10727608

[19] Zeng J Lai C Luo J Li L. Functional investigation and two-sample Mendelian randomization study of neuropathic pain hub genes obtained by WGCNA analysis. Front Neurosci. (2023) 17:1134330. doi: 10.3389/fnins.2023.1134330, PMID: 37123369 PMC10140399

[20] Colaprico A Silva TC Olsen C Garofano L Cava C Garolini D. TCGAbiolinks: an R/Bioconductor package for integrative analysis of TCGA data. Nucleic Acids Res. (2016) 44:e71. doi: 10.1093/nar/gkv1507, PMID: 26704973 PMC4856967

[21] Yan B Ren F Shang W Gong X. Transcriptomic analysis reveals genetic cross-talk between periodontitis and hypothyroidism. Dis Markers. (2022) 2022:5736394. doi: 10.1155/2022/5736394, PMID: 35450027 PMC9017577

[22] Ito K Murphy D. Application of ggplot2 to pharmacometric graphics. CPT Pharmacometrics Syst Pharmacol. (2013) 2:e79. doi: 10.1038/psp.2013.56, PMID: 24132163 PMC3817376

[23] Yu G Wang LG Han Y He QY. clusterProfiler: an R package for comparing biological themes among gene clusters. OMICS. (2012) 16:284–7. doi: 10.1089/omi.2011.0118, PMID: 22455463 PMC3339379

[24] Chen XM Zhao Y Wu XD Wang MJ Yu H Lu JJ. Novel findings from determination of common expressed plasma exosomal microRNAs in patients with psoriatic arthritis, psoriasis vulgaris, rheumatoid arthritis, and gouty arthritis. Discov Med. (2019) 28:47–68., PMID: 31465725

[25] Szklarczyk D Gable AL Lyon D Junge A Wyder S Huerta-Cepas J. STRING v11: protein-protein association networks with increased coverage, supporting functional discovery in genome-wide experimental datasets. Nucleic Acids Res. (2019) 47:D607–d13. doi: 10.1093/nar/gky1131, PMID: 30476243 PMC6323986

[26] Robin X Turck N Hainard A Tiberti N Lisacek F Sanchez JC. pROC: an open-source package for R and S+ to analyze and compare ROC curves. BMC Bioinf. (2011) 12:77. doi: 10.1186/1471-2105-12-77, PMID: 21414208 PMC3068975

[27] Kumar L EF M. Mfuzz: a software package for soft clustering of microarray data. Bioinformation. (2007) 2:5–7. doi: 10.6026/97320630002005, PMID: 18084642 PMC2139991

[28] Shannon P Markiel A Ozier O Baliga NS Wang JT Ramage D. Cytoscape: a software environment for integrated models of biomolecular interaction networks. Genome Res. (2003) 13:2498–504. doi: 10.1101/gr.1239303, PMID: 14597658 PMC403769

[29] Lu MLR De Venecia TA Goyal A Rodriguez Ziccardi M Kanjanahattakij N Shah MK. Psychiatric conditions as predictors of rehospitalization among African American patients hospitalized with heart failure. Clin Cardiol. (2017) 40:1020–5. doi: 10.1002/clc.22760, PMID: 28750156 PMC6490576

[30] Coello K Kjaerstad HL Stanislaus S Melbye S Faurholt-Jepsen M Miskowiak KW. Thirty-year cardiovascular risk score in patients with newly diagnosed bipolar disorder and their unaffected first-degree relatives. Aust N Z J Psychiatry. (2019) 53:651–62. doi: 10.1177/0004867418815987, PMID: 30518229

[31] Russo V Bottino R Rago A Papa AA Liccardo B Proietti R. The effect of sacubitril/valsartan on device detected arrhythmias and electrical parameters among dilated cardiomyopathy patients with reduced ejection fraction and implantable cardioverter defibrillator. J Clin Med. (2020) 9:1111. doi: 10.3390/jcm9041111, PMID: 32294983 PMC7230317

[32] Moore R Moore K Stairley R Fulmer D Guo L Norris R. Abstract MP173: loss of DCHS1 promotes mitral valve prolapse through cytoskeleton destabilization. Circ Res. (2020) 127. doi: 10.1161/res.127.suppl_1.MP173

[33] Han MR Han KM Kim A Kang W Kang Y Kang J. Whole-exome sequencing identifies variants associated with structural MRI markers in patients with bipolar disorders. J Affect Disord. (2019) 249:159–68. doi: 10.1016/j.jad.2019.02.028, PMID: 30772743

[34] Kloiber S Rosenblat JD Husain MI Ortiz A Berk M Quevedo J. Neurodevelopmental pathways in bipolar disorder. Neurosci Biobehav Rev. (2020) 112:213–26. doi: 10.1016/j.neubiorev.2020.02.005, PMID: 32035092

[35] Cappello S Gray MJ Badouel C Lange S Einsiedler M Srour M. Mutations in genes encoding the cadherin receptor-ligand pair DCHS1 and FAT4 disrupt cerebral cortical development. Nat Genet. (2013) 45:1300–8. doi: 10.1038/ng.2765, PMID: 24056717

[36] Kuta A Mao Y Martin T Ferreira de Sousa C Whiting D Zakaria S. Fat4-Dchs1 signalling controls cell proliferation in developing vertebrae. Development. (2016) 143:2367–75. doi: 10.1242/dev.131037, PMID: 27381226 PMC4958319

[37] Mao B Gao Y Bai Y Yuan Z. Hippo signaling in stress response and homeostasis maintenance. Acta Biochim Biophys Sin (Shanghai). (2015) 47:2–9. doi: 10.1093/abbs/gmu109, PMID: 25476206

[38] O’Neill AC Kyrousi C Einsiedler M Burtscher I Drukker M Markie DM. Mob2 insufficiency disrupts neuronal migration in the developing cortex. Front Cell Neurosci. (2018) 12:57. doi: 10.3389/fncel.2018.00057, PMID: 29593499 PMC5857600

[39] Park ES Woods DC Tilly JL. Bone morphogenetic protein 4 promotes mammalian oogonial stem cell differentiation via Smad1/5/8 signaling. Fertil Steril. (2013) 100:1468–75. doi: 10.1016/j.fertnstert.2013.07.1978, PMID: 23993924 PMC4266321

[40] Sun L Sucosky P. Bone morphogenetic protein-4 and transforming growth factor-beta1 mechanisms in acute valvular response to supra-physiologic hemodynamic stresses. World J Cardiol. (2015) 7:331–43. doi: 10.4330/wjc.v7.i6.331, PMID: 26131338 PMC4478568

[41] Guo WT Dong DL. Bone morphogenetic protein-4: a novel therapeutic target for pathological cardiac hypertrophy/heart failure. Heart Fail Rev. (2014) 19:781–8. doi: 10.1007/s10741-014-9429-8, PMID: 24736806

[42] Majerczak J Filipowska J Tylko G Guzik M Karasinski J Piechowicz E. Impact of long-lasting spontaneous physical activity on bone morphogenetic protein 4 in the heart and tibia in murine model of heart failure. Physiol Rep. (2020) 8:e14412. doi: 10.14814/phy2.14412, PMID: 32319199 PMC7174143

[43] Wu X Sagave J Rutkovskiy A Haugen F Baysa A Nygård S. Expression of bone morphogenetic protein 4 and its receptors in the remodeling heart. Life Sci. (2014) 97:145–54. doi: 10.1016/j.lfs.2013.12.030, PMID: 24398041

[44] Huang F Hu L Zhang Y Qu X Xu J. BMP4 moderates glycolysis and regulates activation and interferon-gamma production in CD4+ T cells. Front Immunol. (2021) 12:702211. doi: 10.3389/fimmu.2021.702211, PMID: 34413854 PMC8368433

[45] Saal HM Prows CA Guerreiro I Donlin M Knudson L Sund KL. A mutation in FRIZZLED2 impairs Wnt signaling and causes autosomal dominant omodysplasia. Hum Mol Genet. (2015) 24:3399–409. doi: 10.1093/hmg/ddv088, PMID: 25759469 PMC4834928

[46] Wang P Mokhtari R Pedrosa E Zheng D Lachman H. SU14 - Transcriptome analysis of wnt7a-mediated gene expression in human neural progenitor cells derived from control and chd8 haploinsufficient induced pluripotent stem cells. Eur Neuropsychopharmacol. (2019) 29:S894. doi: 10.1016/j.euroneuro.2017.08.203

[47] Rizig MA McQuillin A Ng A Robinson M Harrison A Zvelebil M. A gene expression and systems pathway analysis of the effects of clozapine compared to haloperidol in the mouse brain implicates susceptibility genes for schizophrenia. J Psychopharmacol. (2012) 26:1218–30. doi: 10.1177/0269881112450780, PMID: 22767372

[48] Zhu X Xu M Leu NA Morrisey EE Millar SE. FZD2 regulates limb development by mediating beta-catenin-dependent and -independent Wnt signaling pathways. Dis Model Mech. (2023) 16., PMID: 36867021 10.1242/dmm.049876 PMC10073008

[49] Leon E Nde C Ray RS Preciado D Zohn IE. ALDH1A2-related disorder: A new genetic syndrome due to alteration of the retinoic acid pathway. Am J Med Genet A. (2023) 191:90–9. doi: 10.1002/ajmg.a.62991, PMID: 36263470 PMC9805811

[50] Steiner MB Vengoechea J Collins RT 2nd. Duplication of the ALDH1A2 gene in association with pentalogy of Cantrell: a case report. J Med Case Rep. (2013) 7:287. doi: 10.1186/1752-1947-7-287, PMID: 24377748 PMC3917519

[51] Beecroft SJ Ayala M McGillivray G Nanda V Agolini E Novelli A. Biallelic hypomorphic variants in ALDH1A2 cause a novel lethal human multiple congenital anomaly syndrome encompassing diaphragmatic, pulmonary, and cardiovascular defects. Hum Mutat. (2021) 42:506–19. doi: 10.1002/humu.24179, PMID: 33565183

[52] Mohamed TM Zi M Prehar S Maqsood A Abou-Leisa R Nguyen L. The tumour suppressor Ras-association domain family protein 1A (RASSF1A) regulates TNF-alpha signalling in cardiomyocytes. Cardiovasc Res. (2014) 103:47–59. doi: 10.1093/cvr/cvu111, PMID: 24776599 PMC4207857

[53] Li H Bian Y. Fibroblast-derived interleukin-6 exacerbates adverse cardiac remodeling after myocardial infarction. Korean J Physiol Pharmacol. (2024) 28:285–94. doi: 10.4196/kjpp.2024.28.3.285, PMID: 38682176 PMC11058547

[54] Andenaes K Lunde IG Mohammadzadeh N Dahl CP Aronsen JM Strand ME. The extracellular matrix proteoglycan fibromodulin is upregulated in clinical and experimental heart failure and affects cardiac remodeling. PloS One. (2018) 13:e0201422. doi: 10.1371/journal.pone.0201422, PMID: 30052659 PMC6063439

[55] Shih YC Chen CL Zhang Y Mellor RL Kanter EM Fang Y. Endoplasmic reticulum protein TXNDC5 augments myocardial fibrosis by facilitating extracellular matrix protein folding and redox-sensitive cardiac fibroblast activation. Circ Res. (2018) 122:1052–68. doi: 10.1161/CIRCRESAHA.117.312130, PMID: 29535165 PMC5899016

[56] Frangogiannis NG. The extracellular matrix in ischemic and nonischemic heart failure. Circ Res. (2019) 125:117–46. doi: 10.1161/CIRCRESAHA.119.311148, PMID: 31219741 PMC6588179

[57] Liu X Li J Yang X Li X Kong J Qi D. Carcinoma-associated fibroblast-derived lysyl oxidase-rich extracellular vesicles mediate collagen crosslinking and promote epithelial-mesenchymal transition via p-FAK/p-paxillin/YAP signaling. Int J Oral Sci. (2023) 15:32. doi: 10.1038/s41368-023-00236-1, PMID: 37532712 PMC10397209

[58] Dityatev A Seidenbecher C Morawski M. Brain extracellular matrix: An upcoming target in neurological and psychiatric disorders. Eur J Neurosci. (2021) 53:3807–10. doi: 10.1111/ejn.15336, PMID: 34077569

[59] Wlodarczyk J Mukhina I Kaczmarek L Dityatev A. Extracellular matrix molecules, their receptors, and secreted proteases in synaptic plasticity. Dev Neurobiol. (2011) 71:1040–53. doi: 10.1002/dneu.20958, PMID: 21793226

[60] Dzyubenko E Hermann DM. Neuroglia and extracellular matrix molecules. Handb Clin Neurol. (2025) 209:197–211. doi: 10.1016/B978-0-443-19104-6.00010-3, PMID: 40122625

[61] Mitterauer BJ. Downregulation and upregulation of glial connexins may cause synaptic imbalances responsible for the pathophysiology of bipolar disorder. CNS Neurosci Ther. (2011) 17:281–93. doi: 10.1111/j.1755-5949.2010.00178.x, PMID: 20626435 PMC6493903

[62] Piletic K Kunej T. MicroRNA epigenetic signatures in human disease. Arch Toxicol. (2016) 90:2405–19. doi: 10.1007/s00204-016-1815-7, PMID: 27557899

[63] Guo Y Ding X Dai C Wang W Chen J Chen S. miR-1343-3p inhibits autophagy by directly targeting ATG7 in multiple myeloma cells. Biomed Rep. (2024) 21:185. doi: 10.3892/br.2024.1873, PMID: 39420924 PMC11484188

[64] Bielawska M Warszynska M Stefanska M Blyszczuk P. Autophagy in heart failure: insights into mechanisms and therapeutic implications. J Cardiovasc Dev Dis. (2023) 10:352. doi: 10.3390/jcdd10080352, PMID: 37623365 PMC10456056

[65] Berk M Dodd S Kauer-Sant’anna M Malhi GS Bourin M Kapczinski F. Dopamine dysregulation syndrome: implications for a dopamine hypothesis of bipolar disorder. Acta Psychiatr Scand Suppl. (2007) 116:41–9. doi: 10.1111/j.1600-0447.2007.01058.x, PMID: 17688462

[66] Rawani NS Chan AW Dursun SM Baker GB. The underlying neurobiological mechanisms of psychosis: focus on neurotransmission dysregulation, neuroinflammation, oxidative stress, and mitochondrial dysfunction. Antioxidants (Basel). (2024) 13:709. doi: 10.3390/antiox13060709, PMID: 38929148 PMC11200831

[67] Zhan L Kerr JR Lafuente MJ Maclean A Chibalina MV Liu B. Altered expression and coregulation of dopamine signalling genes in schizophrenia and bipolar disorder. Neuropathol Appl Neurobiol. (2011) 37:206–19. doi: 10.1111/j.1365-2990.2010.01128.x, PMID: 20874815

[68] Sturm B Pacher R Strametz-Juranek J Berger R Frey B Stanek B. Effect of beta 1 blockade with atenolol on progression of heart failure in patients pretreated with high-dose enalapril. Eur J Heart Fail. (2000) 2:407–12. doi: 10.1016/S1388-9842(00)00120-3, PMID: 11113718

[69] Yusuf S Joseph P Dans A Gao P Teo K Xavier D. Polypill with or without Aspirin in Persons without Cardiovascular Disease. N Engl J Med. (2021) 384:216–28. doi: 10.1056/NEJMoa2028220, PMID: 33186492 PMC7116860

[70] Luo J Li L Zeng Q. Effects of dapagliflozin in combination with hydrochlorothiazide on cardiac remodeling in rats with heart failure after myocardial infarction. Int Heart J. (2024) 65:913–28. doi: 10.1536/ihj.24-069, PMID: 39343595

