# Supplementary material for: Preliminary exploration of potential biomarkers for heart failure and bipolar disorder: an exploratory study based on bioinformatics
Source: Front Psychiatry. 2025 Sep 10;16:1627105. doi: 10.3389/fpsyt.2025.1627105 (PMC12459720; doi:10.3389/fpsyt.2025.1627105)
Supplement: Supplementary file 1 [file Supplementaryfile1.pdf]

Supplementary Material

1 Supplementary Figures and Tables

1.1 Supplementary Figures

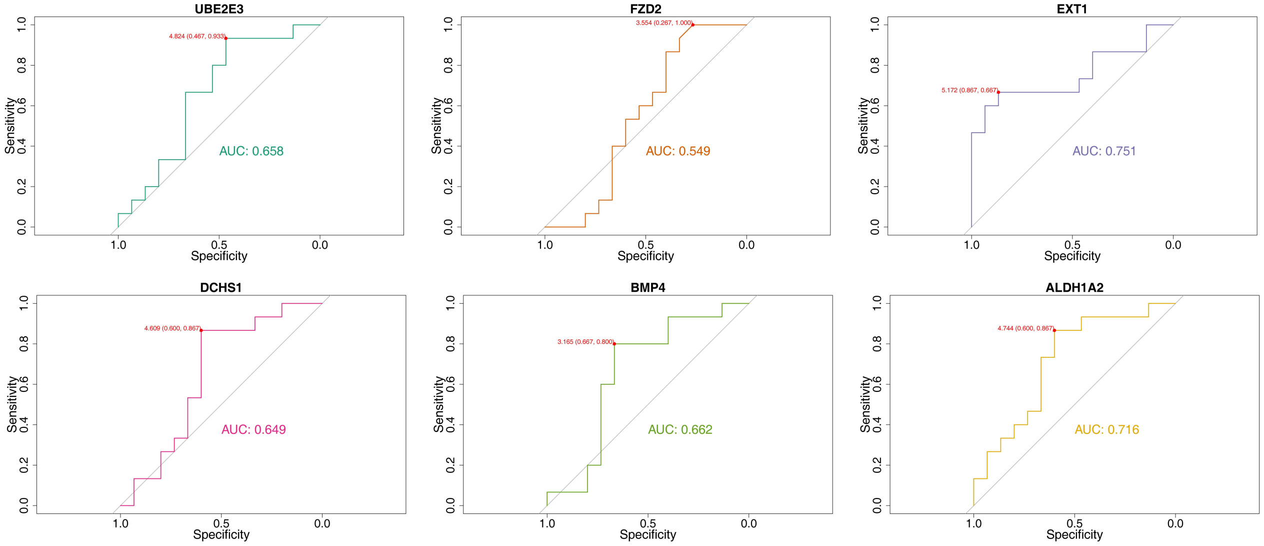

Supplementary Figure 1. ROC curve analysis of biomarkers in GSE16499.

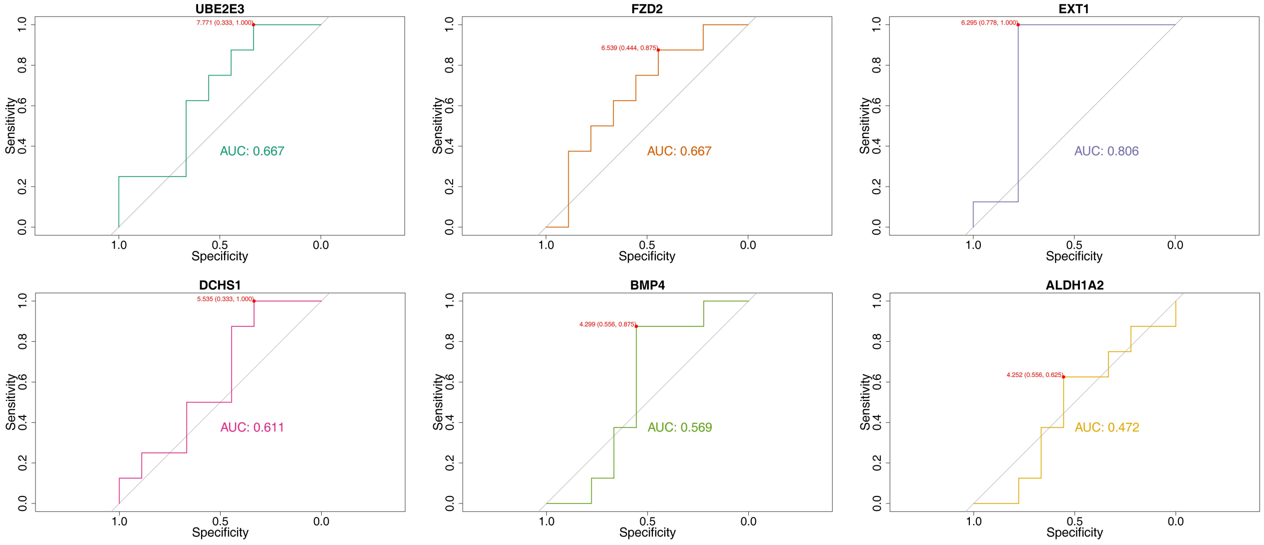

Supplementary Figure 2. ROC curve analysis of biomarkers in GSE18312.

1.2 Supplementary Table

Supplementary Table 1 Drug information.

Gene	Drug	Status	Class	Interaction score
FZD2	VANTICTUMAB	investigational	Not Available	0.5800422062098392
ALDH1A2	RETINOL	Approved,Nutraceutical,Vet approved	Substrate,Inducer,Binder,Ligand	1.160084412419678
ALDH1A2	HYDROCHLOROTHIAZIDE	Approved,Vet approved	Inhibitor,Substrate,Binder	0.4539460744250916
ALDH1A2	ATENOLOL	Approved	Antagonist,Substrate	3.728842754206109
